# Therapeutic Potential of Fungal Terpenes and Terpenoids: Application in Skin Diseases

**DOI:** 10.3390/molecules29051183

**Published:** 2024-03-06

**Authors:** Monika Trepa, Katarzyna Sułkowska-Ziaja, Katarzyna Kała, Bożena Muszyńska

**Affiliations:** 1 Department of Pharmaceutical Botany, Faculty of Pharmacy, Collegium Medicum, Jagiellonian University, 9 Medyczna St., 30-688 Kraków, Poland; monika.trepa@doctoral.uj.edu.pl (M.T.); k.kala@uj.edu.pl (K.K.); bozena.muszynska@uj.edu.pl (B.M.); 2 Doctoral School of Medical and Health Sciences, Collegium Medicum, Jagiellonian University, 16 Św. Łazarza St., 30-530 Kraków, Poland

**Keywords:** fungal terpenes and terpenoids classification, skin diseases, skin cancer, anti-inflammatory, inhibition of tyrosinase

## Abstract

Terpenes and their derivatives comprise a diverse group of natural compounds with versatile medicinal properties. This article elucidates the general characteristics of fungal terpenes and terpenoids, encompassing their structure and biogenesis. The focal point of this work involves a comprehensive overview of these compounds, highlighting their therapeutic properties, mechanisms of action, and potential applications in treating specific skin conditions. Numerous isolated terpenes and terpenoids have demonstrated noteworthy anti-inflammatory and anti-microbial effects, rivalling or surpassing the efficacy of currently employed treatments for inflammation or skin infections. Due to their well-documented antioxidant and anti-cancer attributes, these compounds exhibit promise in both preventing and treating skin cancer. Terpenes and terpenoids sourced from fungi display the capability to inhibit tyrosinase, suggesting potential applications in addressing skin pigmentation disorders and cancers linked to melanogenesis dysfunctions. This paper further disseminates the findings of clinical and in vivo research on fungal terpenes and terpenoids conducted thus far.

## 1. Introduction

Fungi represent a valuable reservoir of bioactive molecules, serving as secondary metabolites with promising therapeutic attributes [1]. These molecules encompass various compounds, including terpenes and terpenoids, which are distinguished by their diverse chemical structures [2]. The heterogeneity within terpene compound classes contributes to a wide array of biological activities. From the well-established anti-cancer and anti-inflammatory properties of sesquiterpenes to the anti-microbial activity of monoterpenes, the chemical diversity exhibited by fungal terpenes and terpenoids provides a versatile toolkit for potential therapeutic applications [3].

The skin, being the largest human organ, plays a pivotal role as a barrier against ex-ternal environmental factors and potential pathogens [4]. Conventional treatments often come with limitations such as side effects and incomplete efficacy. In response to these challenges, there has been a heightened search for alternative and complementary therapeutic agents, with a growing focus on natural compounds [5].

Fungi, acknowledged as a valuable source of bioactive compounds, have been integral to traditional medicine for centuries [6]. Advances in analytical techniques and pharmacological studies have revealed the presence of numerous terpenes in various fungal species [7]. Terpenes and terpenoids, distinguished by their unique chemical structures and diverse biological activities, have shown promise in modulating cellular processes, including anti-inflammatory, anti-oxidant, and immunomodulatory effects [8,9]. Moreover, these compounds have exhibited anti-oxidant properties, offering potential protection against oxidative stress—a prevalent factor in skin aging and certain dermatoses [10]. These properties make fungal terpenoids appealing candidates for developing therapeutic interventions in dermatology [11]. Understanding potential mechanisms of action is pivotal for recognizing the therapeutic significance of fungal terpenoids in dermatology. These compounds possess the ability to modulate cell signaling pathways, influence gene expression, and interact with key molecular targets involved in skin homeostasis. The specific mechanisms governing the impact of fungal terpenes and their derivatives on inflammatory responses, wound healing, and other dermatological processes are active areas of research that could contribute to the development of targeted therapies [12].

This review aims to provide a comprehensive overview of the various classes of terpenes and terpenoids isolated from fungi and their potential mechanisms of action in the context of skin diseases. The documented properties and efficacy of these compounds, particularly when compared to existing treatments, indicate promising opportunities for the development of new therapeutic strategies for a wide range of skin diseases. This article primarily analyzes compounds isolated from the fruiting bodies of various species within the Ascomycota and Basidiomycota phyla, as well as examples of compounds from filamentous fungi (mainly Ascomycota).

## 2. General Characteristics and Occurrence of Terpenes and Terpenoids

Terpenoids, also referred to as isoprenoids, are organic chemical compounds produced by many plants, fungi, animals, bacteria, and other microbes [13]. Coined by Dumas in 1866, the term “terpene” originates from the Latin word “turpentine” (*Balsamum terebinthinae*) [14]. In nature, the term “terpene” is employed to characterize compounds composed of isoprene units [15]. The varied structures and functions of terpenes and their derivatives position them as subjects of research for commercial applications. These compounds have been substantiated to possess anti-cancer, anti-microbial, anti-fungal, anti-parasitic, anti-viral, anti-allergic, anti-spasmodic, anti-hyperglycemic, anti-inflammatory, and immunomodulatory effects. Additionally, they serve as natural insecticides and find utility as protective agents in the storage of agricultural products [16]. Terpenes and terpenoids have applications in dermatology and the cosmetic industry as components in skincare products owing to their diverse therapeutic actions. Their antioxidant properties contribute to safeguarding skin cells from aging and UV-induced damage, while also hindering melanogenesis. Compounds such as retinoids, vitamin A metabolites, and various forms of vitamin E can counteract photoaging by modulating epidermal keratinization, inhibiting UV-induced matrix metalloproteinases, and suppressing pigmentation through the inhibition of the tyrosinase enzyme in melanin synthesis. Orally administered carotenoids, vitamin E, and other terpenoids like carnosic acid, squalene, and coenzyme Q10 are utilized in functional foods and supplements to enhance skin condition and overall health benefits [17]. Terpenoids extracted from fungal species have exhibited anti-inflammatory effects by reducing nitric oxide (NO) and pro-inflammatory cytokines such as IL-1β, IL-6, and TNF-α. Additionally, they demonstrate anti-microbial effects against pathogens like *Escherichia coli*, *Staphylococcus aureus*, and *Bacillus cereus* [18,19,20]. With their advantageous properties, terpenes and their derivatives hold promise for the treatment of various skin conditions, prompting further research to enhance understanding and practical applications in dermatological therapy.

## 3. Biogenesis

Biogenetically, terpenes originate from isopentenyl diphosphate or its isomer dimethylallyldiphosphate [21]. In 1887, chemist Otto Wallach first proposed the hypothesis that terpenes result from the polymerization of pentene (C_5_H_8_), also known as isoprene. The biosynthesis of terpenes begins with three acetyl-CoA units that generate mevalonic acid, serving as the precursor for isopentenyl diphosphate (IPP) and its isomer dimethylallyl diphosphate (DMAPP) [14]. Haemiterpenes, the simplest compounds in the terpene group, directly originate from IPPs, DMAPPs, or one of the intermediates in their biosynthetic pathway, such as mevalonic acid [22]. The synthesis of monoterpenes initiates with the initial compound geranyl diphosphate (GPP), which forms through the phosphorylation of mevalonic acid [15]. GPP can combine with IPP to produce farnesyl diphosphate (FPP), the initial compound for sesquiterpene synthesis. In the next step, FPP can condense with the IPP molecule to form geranyl-geranyl-PP (GGPP), a diterpene. The dimerization of two GGPPs then yields 16-trans-phytoene, a tetraterpene, from which carotenoids are synthesized. Condensation of two FPP molecules generates squalene, a precursor of triterpenoids, crucial to the synthesis of triterpenes and sterols [15,22].

All fungal terpenoid products originate from IPPs and DMAPPs, synthesized through the mevalonate pathway [23]. The formation of individual compounds primarily occurs through the transformation of the starting compounds via processes such as cyclization, oxidation, attachment, or de-substitution [21]. The significant diversity of terpenoids arises from the existence of numerous distinct terpene synthases, with some of them generating multiple by-products [14].

## 4. Structure and Chemical Classification of Terpenes and Terpenoids

In 1953, Leopold Ružička formulated the isoprene principle, asserting that the carbon skeleton of terpenes consists of isoprene units linked by regular (head-tail) and irregular arrangements [24]. Terpenes can be classified into different classes based on the number of isoprene units (*n*) in the molecule: hemiterpenes (C_5_H_8_), monoterpenes (C_10_H_16_), sesquiterpenes (C_15_H_24_), diterpenes (C_20_H_32_), triterpenes (C_30_H_48_), tetraterpenes (C_40_H_64_), and polyterpenes (C_5_H_8_)*_n_*. It is essential to note that terpenoids differ from terpenes; terpenes are simple, unsaturated hydrocarbons polymerized by isoprene units, while terpenoids belong to terpene derivatives with attached elements or functional groups, such as oxidized and nitrogenated branches, as well as methyl oxidized groups that can be removed and moved at different positions [25,26].

### 4.1. Haemiterpens

Haemiterpenes are compounds consisting of a single isoprene molecule. There are fewer than 100 known haemiterpenes. The best-known is isoprene, which is not found in nature. Natural haemiterpenes include tiglic acid and caffeic acid [15,27].

### 4.2. Monoterpenes and Monoterpenoids

Monoterpenes and monoterpenoids comprise a substantial group of organic compounds, each composed of two isoprene units. Monoterpenes consist solely of carbon and hydrogen atoms, with the molecular formula C_10_H_16_, whereas monoterpenoids are modified terpenes with various functional groups, resulting in the formation of alcohols, carboxylic acids, ketones, aldehydes, and phenols [28]. There are more than 30 known carbon skeletons of monoterpenes, with approximately 20 being common and categorizable into acyclic, monocyclic, bicyclic, and tricyclic types [27]. Among the acyclic monoterpenoids, alcohols and aldehydes are predominantly found. Examples of this group include linalool and geraniol. Monocyclic monoterpenes consist of a single cyclic system, such as cyclopropane, cyclobutane, and cyclohexane. They can exist as hydrocarbons or undergo reactions like carboxylation and oxidation to form compounds of the alcohol, ketone, or oxide group. Well-known monocyclic monoterpenoids include limonene, terpinene, and terpineol. Bicyclic monoterpenes contain two linked cyclic systems. Through chemical reactions, they primarily form compounds from the hydrocarbons, alcohols, and ketones groups. Representatives of bicyclic monoterpenoids include α- and β-pinene, fenchone, and α-thujene [21].

Various monoterpenes and their derivatives have been identified in fungal fruiting bodies, with examples including linalool in *Ganoderma lucidum*, *Hericium erinaceus*, and *Fomitopsis betulina*; limonene in *G. lucidum*, *H. erinaceus*, and *Lentinula edodes*; *α*-terpineol in *Antrodia camphorata*, *G. lucidum*, *F. betulina*, *Pleurotus eryngi*, and *β*-terpineol in *A. camphorata* and *H. erinaceus* [29]. Recently, new monoterpenoids have been isolated, such as 4-hydroxy-4-isopropenyl-cyclohexanomethanol acetate of the menthane type from *Craterellus cornucopioides* and 6,7,8-trihydroxy-2,6-dimethyl octanoic acid from the poisonous fungus *Trogia venenata* [30,31].

### 4.3. Sesquiterpenes and Sesquiterpenoids

Sesquiterpenes and sesquiterpenoids constitute a group of 15-carbon compounds formed by the combination of 3 isoprene units, with the basic molecular formula C_15_H_24_ [32]. These compounds can take on various structures, including linear, cyclic, bicyclic, tricyclic, and tetracyclic systems. Except for simple farnesane and some irregular acyclic sesquiterpenoids, most exhibit cyclic skeletons. They undergo numerous modifications, creating not only hydrocarbons but also oxidized forms such as lactones, alcohols, acids, aldehydes, and ketones. Moreover, they can occur at different oxidation and reduction levels, forming isomers. Well-known compounds in this group include bisabolol, bisabolene, and farnesene [21].

Numerous sesquiterpenes and their derivatives have been extracted from various fungal species. Compounds such as aristolane, bisabolane, kuparene, drimane, phomannozane, lactarate, nordazinane, and sterpurane were acquired from the fruiting bodies of species such as *Stereum hirsutum*, *Inonotus rickii*, *Pleurotus cornucopiae*, *Anthracophyllum* sp., *Agrocybe salicacola*, *Neonothopanus nambi*, *Russula lepida*, *Russula amarissima*, *Strobilurus ohshimae*, *Lactarius vellereus*, and *Lacterius subpiperatus* [33]. In a recent investigation, numerous novel sesquiterpene-derived cuparans were isolated from the fruiting bodies of *Flammulina velutipes* [34]. Conversely, bergamotene, selinene, and santalene have been identified in the species *Inonotus obliquus.* A new trichothecene sesquiterpenoid and tremulane sesquiterpenoid were isolated from *Gymnopilus junonius* [35]. The sesquiterpenoid phallacic acid A was isolated from the *Phallus luteus* species, and its chemical structure was established [36]. Previously undescribed compounds named incarnatins A-D, incarnolactones A-C, incarnatin E, incarnetic acid, and incarnanin were isolated from *Gloeostereum incarnatum* [19].

### 4.4. Diterpenes and Diterpenoids

Diterpenes and diterpenoids are compounds consisting of four isoprene units with the basic formula C_20_H_32_ [37]. Similar to monoterpenes and sesquiterpenes, diterpenoids can form linear, bicyclic, tricyclic, and multi-cyclic structures. These compounds can be categorized as either hydrophilic or lipophilic diterpenoids, depending on the substituents attached to them. In nature, diterpenoids are commonly found in polyoxygenated forms, featuring ketone and hydroxyl groups, and they are often esterified with aliphatic or aromatic acids. Some diterpenoids can undergo glycosylation reactions, where they bind with sugar molecules to form glycosides. Examples of diterpenoids include vitamin A, gibberellic acid, carnosolic acid, and abietanoic acid [21]. A group of diterpenoids commonly found in fungi includes tetracyclic gibberellins, such as gibberellic acid, which is isolated from *Gibberella fujikuroi* [15]. Another group of kjatan-type diterpenoids was isolated in the fruiting bodies of fungi belonging to the phylum Basidiomycota. These compounds are present, among others, in the fruiting bodies of *Cyathus helenae*, *Cyathus africanus*, *Cyathus earlei*, *Cyathus striatius*, *H. erinaceus*, *Sarcodon scabrosus*, *Sarcodon glaucopus*, *Sarcodon cyrneus*, and *Strobilurus tenacellus* [38].

### 4.5. Sesterpenes and Sesterpenoids

Sesterpenes consist of 25 carbon atoms with 5 isoprene units, represented by the molecular formula C_25_H_40_. They exist in various forms, including linear, monocyclic, bicyclic, tricyclic, tetracyclic, and macrocyclic structures. These compounds occur naturally, primarily in fungi, marine organisms, insects and their protective waxes, sponges, lichens, and less frequently in plants [15,27]. Representatives of sesterpenoids include sesterstatin, leucosceptrin, psylostachin, and heliocide H [27].

Examples of sesterpenes isolated from fungal species include ophiobolins, extracted from *Ophiobolus heterostrophus*, *Ophiobolus miyabeanus*, and *Aspergillus ustus* [3]. The newly discovered sesterpenoids in fungi are secoemestrin D and emericellenes A-E found in *Emericella* sp. [39]. The presence of compounds from this group has also been reported in species such as *Pleurotus ostreatus* and *Scleroderma areolatum* [40].

### 4.6. Triterpenes and Triterpenoids

Triterpenes consist of six isoprene units with the basic molecular formula C_30_H_48_. They primarily exist as cyclic structures, although exceptions like squalene and squalane have linear structures. Triterpenoids typically form relatively complex cyclic structures, mostly five- to rarely four-cyclic, and are predominantly alcohols, aldehydes, or carboxylic acids. Through glycosylation reactions, they can combine with sugar molecules to form saponins. Triterpenes and their derivatives are found in significant quantities in plants, fungi, lichens, and algae [15]. Examples of triterpenoids include betulin, fusidic, helvolic, and ursolic acid [27].

**Figure 1:**
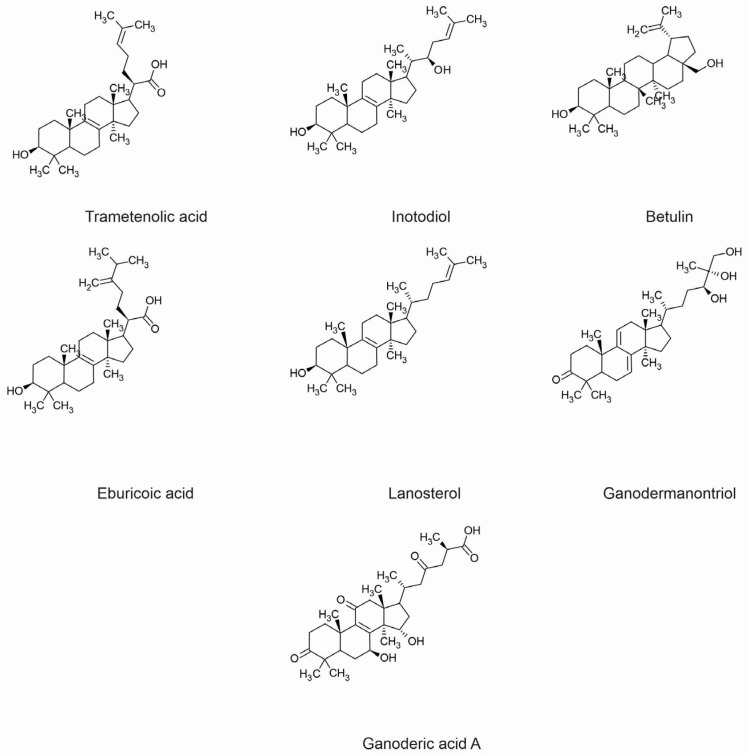
Chemical structures of selected fungal terpenoids with applications in the prevention and treatment of skin diseases.

So far, numerous triterpenes and triterpenoids have been isolated from fungal species. A four-cyclic protostane and its derivatives have been extracted from *Cephalosporium caerulens*. Others in this category, such as helvolic acid (*Aspergillus fumigatus*) and fusidic acid (*Fusidium coccineum*), are well-known anti-microbial agents used in treating skin infections [15]. Ganoderic acids, obtained from *G. lucidum*, serve as examples of tetracyclic triterpenoids of the lanostane type (Figure 1) [15].

### 4.7. Tetraterpenes and Tetraterpenoids

Tetraterpenes are composed of eight isoprene units with the molecular formula C_40_H_64_. The most well-known tetraterpenoids are the carotenoid hydrocarbons, which are natural fat-soluble pigments, and their oxygen analogs, the xanthophylls and their esters. Examples of carotenoid hydrocarbons include lycopene, while xanthophylls include lutein and zeaxanthin [21]. Combined forms of carotenoids are typically xanthophylls esterified with fatty acid residues, such as palmitic, oleic, or linoleic acids [27]. Tetraterpenoids have also been isolated from fungi, plants, and bacteria [3].

In the case of fungal species, an example of an isolated tetraterpenoid is red *γ*-carotene, extracted in significant quantities from *Penicillium sclerotiorum* [15].

### 4.8. Polyterpenoids

Polyterpenoids are polymeric isoprenoid hydrocarbons comprising more than eight isoprene units. Polyisoprene chains may adopt either *cis* or *trans* conformations. They include rubber, gutta-percha, and betulaprenols. To date, these have not been identified in fungi [15,27].

## 5. Pharmacological Activities in Skin Disorders

Many pharmacological activities exhibited by fungal terpenes and their derivatives have been proven. Several of them demonstrate significant properties for the treatment of dermatological diseases. The individual pharmacological properties of specific fungal terpenes and terpenoids are discussed below, along with their mechanisms of action and potential use in treating skin diseases. The chemical structures of selected compounds with applications in the prevention and treatment of skin diseases are shown in Figure 1.

### 5.1. Anti-Inflammatory Activity

**Figure 2:**
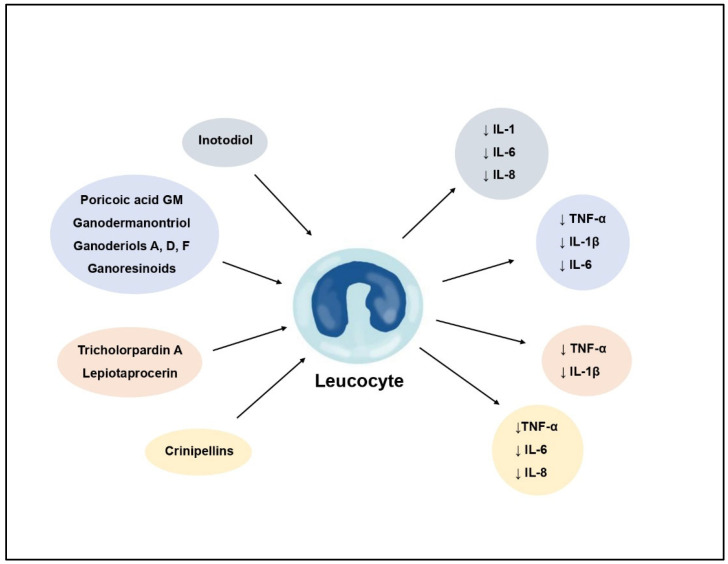
Inhibition of pro-inflammatory cytokines by fungal terpenes and terpenoids.

Inflammation is the body’s immunological response to harmful agents, and cutaneous inflammation is a prevalent symptom in the progression of various dermatological conditions. The development of dermatitis is characterized by redness, pain, burning, and swelling. This dermatosis can result from bacterial and viral infections, allergic reactions, as well as chronic skin diseases such as psoriasis, atopic dermatitis, and acne. Conventional pharmaceutical interventions for inflammatory skin disorders often involve the use of steroids in the form of topical ointments and creams. Despite their notable efficacy, prolonged use is associated with a range of adverse effects. As a result, there is a concerted effort in the ongoing pursuit of novel compounds that demonstrate comparable efficacy in mitigating skin inflammation while minimizing associated side effects. Numerous terpenoid compounds have shown anti-inflammatory properties, exerting their effects through various mechanisms of action. These mechanisms primarily involve the inhibition of pro-inflammatory cytokine expression, modulation of intracellular and extracellular signaling pathways, and the restraint of protein kinases implicated in inflammatory processes (Figure 2).

Inotodiol, a representative of lanostane-type triterpenoid found in Chaga, was investigated for its impact on keratinocytes exposed to UV radiation and TNF-α factor treatment, both known to induce the secretion of pro-inflammatory factors. In both scenarios, the triterpenoid markedly reduced the expression of cytokines (IL-1, IL-6, IL-8) in keratinocytes. The introduction of inotodiol concentrate at concentrations exceeding 2.2 µg/mL led to a decrease in the concentration of the tested pro-inflammatory factors in UV-exposed epidermal cells to baseline levels [11].

**Figure 3:**
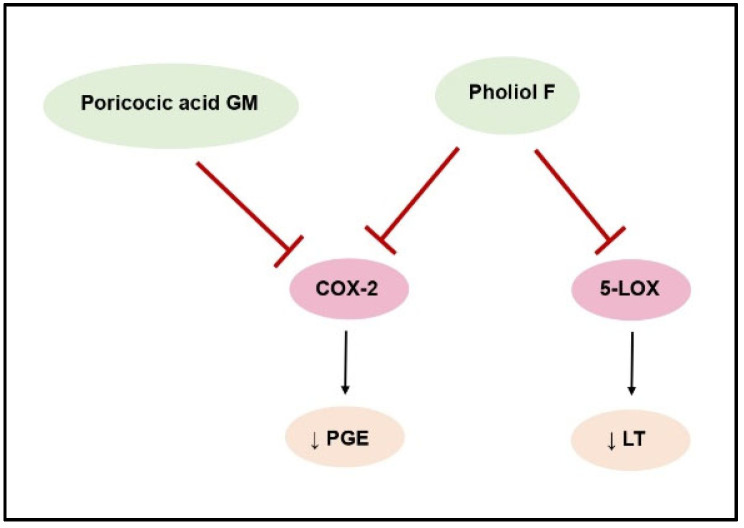
COX-2 and 5-LOX inhibition by fungal terpenoids.

Triterpenes obtained from *Pholiota populnea*, specifically pholiols E-K, underwent evaluation for their ability to inhibit cyclooxygenase 1 (COX-1) and 2 (COX-2), as well as lipoxygenase (5-LOX and 15-LOX) enzymes. Notably, pholiol F demonstrated the most significant inhibitory effect, suppressing COX-2 and 5-LOX activities (Figure 3). This resulted in a reduction in the synthesis of leukotrienes and prostaglandins [41].

**Figure 4:**
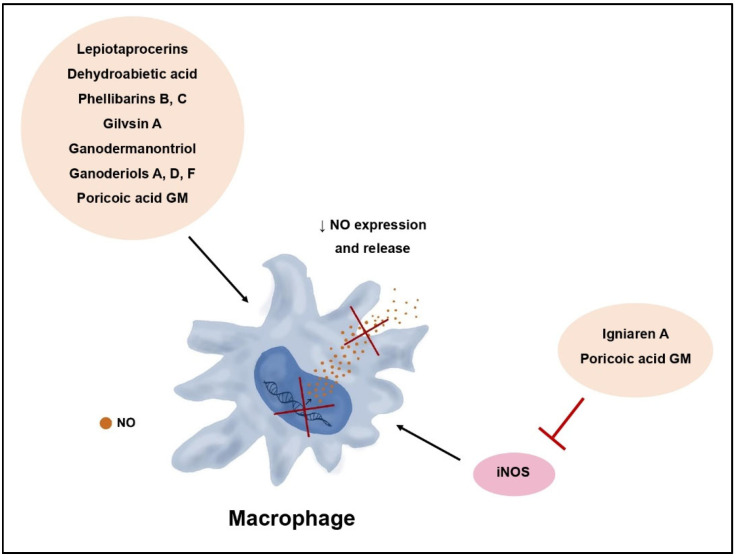
Inhibition of iNOS enzymes and pro-inflammatory nitric oxide (NO) expression and secretion by fungal terpenoids.

Certain terpenes and terpenoids exhibit anti-inflammatory effects by inhibiting the expression and secretion of pro-inflammatory nitric oxide (NO) (Figure 4). Lanostane-type triterpenoids, specifically lepiotaprocerins isolated from *Macrolepiota procera*, serve as examples of compounds that significantly reduce NO production in RAW 264 cells [42]. Inhibitory effects on NO secretion in LPS-induced cells have also been demonstrated for the diterpenoid dehydroabietanoic acid (*Phellinus pini*), as well as phellibarin B, C, and gilvinin A (*Phellinus rhabarbarinus*). Triterpenoids isolated from *Fomitopsis pinicola* impede the NO release from macrophages [43]. On the other hand, lanostane-type triterpenoids, such as igniarene B extracted from *Phellinus igniarius*, significantly inhibit inducible nitric oxide synthase (iNOS) enzymes responsible for regulating macrophage function [44].

Tricholopardin A, identified in the fruiting bodies of *Tricholoma pardinum*, exhibits a robust inhibitory effect on NO production in RAW 264.7 cells. Furthermore, studies have demonstrated its impact on the secretion of pro-inflammatory factors such as PGE2, IL-1β, and TNF-α [45]. A similar effect has also been observed for triterpenoids derived from *Trametes versicolor* and lepiotaprocerins (lanostane-type triterpenoids) isolated from the fruiting bodies of *M. procera* [42,46].

**Figure 5:**
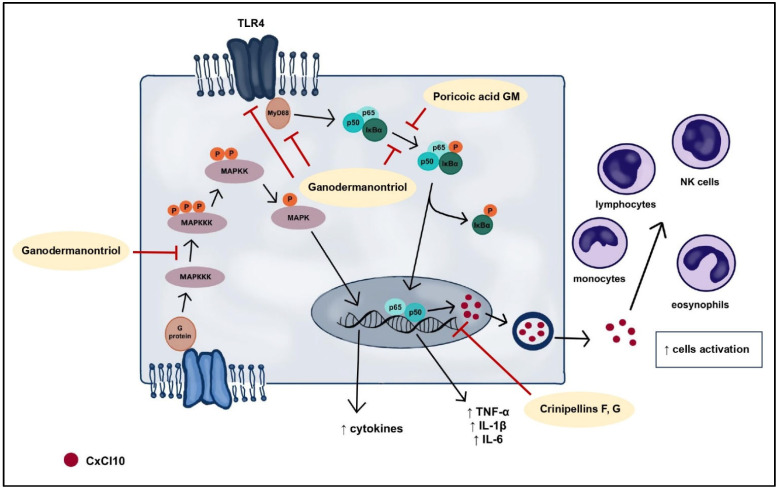
Anti-inflammatory effects of fungal terpenes and terpenoids through effects on MAPK and NF-κB signaling pathways.

Triterpenes derived from *G. lucidum* are anti-inflammatory compounds that exert their effects through multiple mechanisms. Ganodermanontriol (Figure 1) and ganoderiols A, D, F efficiently decrease the production of pro-inflammatory factors, including NO, TNF-α, IL-1β, and IL-6. The former compound has demonstrated the inhibition of the phosphorylation of p65 and IκBα, thereby obstructing the NF-κB signaling pathway, which is implicated in the transcription of pro-inflammatory enzymes and cytokines. Ganodermanontriol also inhibits specific phosphorylated protein kinases within the MAPK pathways, responsible for inducing inflammation. Additionally, it diminishes the expression of the TLR4 protein, a pivotal activator of the NF-κB and MAPK pathways, along with the level of the adaptor molecule MyD88. This binding to TLR4 represents a crucial step in the activation of the signaling cascade and the initiation of the inflammatory response (Figure 5) [47].

Lanostane-type triterpenoids isolated from the *Wolfiporia cocos* also exhibit a similar, dual mechanism of action. In studies assessing their anti-inflammatory properties, poricoic acid GM demonstrated the most potent effect by reducing the release of pro-inflammatory cytokines, including IL-1β, IL-6, TNF-α, and PGE2. Moreover, it suppressed iNOS protein expression and NO production in LPS-induced RAW 264.7 cells, as well as COX-2 expression. This anti-inflammatory effect of poricoic acid GM is ascribed to the inhibition of IκBα phosphorylation, resulting in the blockade of the NF-κB signaling pathway (Figure 5) [48].

An anti-inflammatory mechanism of action, centered on the inhibition of the TLR4/NF-κB and MAPK pathways, has also been exhibited for ganoresinoids—triterpenoids present in *Ganoderma resinaceum*. Similar to ganodermanontriol and poricoic acid GM, the blockade of these signaling pathways led to a decrease in the secretion of pro-inflammatory cytokines, including IL-1β, IL-6, and TNF-α, in LPS-treated cells [49].

The anti-inflammatory impact of terpenoids may arise from the inhibition of CxCl10 chemokine expression, as evidenced by studies on the diterpenoids crinipellins E-H extracted from *Crinipellis* sp. (Figure 5). CxCl10, a small protein orchestrating inflammatory responses by activating monocytes, eosinophils, T lymphocytes, and NK cells, was notably affected. The research affirmed that crinipellin F and crinipellin G significantly diminished chemokine expression in a concentration-dependent manner. Moreover, these compounds hindered NF-κB and interleukin-8 expression. The suppression of the NF-κB signaling pathway likely contributed to the reduction in chemokine levels, given its role as a primary regulator of cxcl10 gene expression. Additionally, PCR analysis revealed that the tested compounds substantially decreased mRNA levels of pro-inflammatory factors like IL-6, IL-8, TNF-α, CCL2, and COX-2, curbing the synthesis of these substances and the chemokines CxCl1, CxCl11, CCL3, crucial in the activation and recruitment of inflammatory cells [50].

Anti-inflammatory triterpenes and their derivatives identified in *Ganoderma* sp. underwent in silico screening to assess their affinity for GR and MCR receptors, the binding sites for commonly used corticosteroids in dermatology. The study revealed that certain compounds displayed substantial affinity and binding energies to GR receptors, comparable to dexamethasone, their natural ligand. However, a lower affinity was observed for MCR receptors. This outcome from the analysis could partially elucidate the robust anti-inflammatory characteristics of ganoderic triterpenes [51].

The diverse range of mechanisms they exhibit suggests that the inhibition of inflammation may occur at multiple points, positioning these compounds as a promising avenue for future therapeutic interventions in inflammatory skin conditions.

*G. lucidum* stands out as a species of particular interest in addressing inflammatory skin conditions, owing to its possession of triterpenes and triterpenoids with well-established and substantial anti-inflammatory effects. A study investigating the skin permeability of components from *G. lucidum* extracts indicated high permeability for triterpenes and triterpenoids, and low permeability for phenols. This observation suggests that terpene compounds might be the primary contributors to the therapeutic effects [52].

*G. lucidum* extracts are currently integrated into dermocosmetics to provide supportive care for conditions like eczema, dermatitis, burns, and inflammatory skin reactions following chemotherapy. These extracts serve as anti-inflammatory and healing ingredients [53]. In a mouse study, triterpene acids—specifically ganoderic and lucidenic acids—demonstrated effectiveness against inflammation when applied in a quantity of 1 µg [54].

A study involving mice with induced ear inflammation examined the anti-inflammatory properties of lanostane-type triterpenoids isolated from the species *F. betulina*. The constituents were applied at a dose of 400 nmol per ear, resulting in a significant reduction in swelling. Notably, some tested substances, particularly polyporenoic acid A and its three derivatives, exhibited greater efficacy compared to the positive control treated with indomethacin [55].

The anti-inflammatory and analgesic properties of *A. camphorata* terpenoids, specifically eburicoic acid (Figure 1) and dehydroeburicoic acid, were evaluated in mice with induced paw inflammation. The compounds were administered at concentrations of 1, 5, and 10 mg/kg body weight, with indomethacin at 10 mg/kg body weight serving as a control. Both test compounds exhibited significant reductions in pain sensation and inflammatory swelling. The 10 mg/kg dose demonstrated efficacy comparable to indomethacin in terms of both analgesic and anti-inflammatory effects. Histopathological examination of inflamed tissues treated with terpenoids revealed a significant decrease in pro-inflammatory cytokines, such as TNF-α and IL-1β [56].

Chemoprevention emerges as a viable strategy to reduce the likelihood of developing cancer. In recent decades, scientists have recognized the pivotal role of chronic inflammation in cancer development. Inotilone, present in the *Inonotus* sp., has been noted for its anti-inflammatory properties under laboratory conditions. In the study, inotilone was topically applied to the skin of mice 30 min prior to TPA treatment. The results showed that inotilone inhibited the production of inflammatory mediators by attenuating nuclear factor-κB (NF-κB) activation and CCAAT/enhancer β-binding protein (C/EBPβ) expression [57].

### 5.2. Antimicrobial Activity

One of the challenges in modern medicine is the escalating resistance of microorganisms to commonly used antibiotics. The dwindling therapeutic options emphasize the active pursuit of new compounds with anti-bacterial, anti-viral, and anti-fungal properties. Numerous studies have explored the anti-microbial properties of fungal terpenes, revealing intriguing activities, particularly in the context of skin infections.

Bacterial skin infections can emerge as primary or secondary infections resulting from concurrent inflammation, fungal infections, injury, or bites. Common bacterial strains responsible for most infections include *Staphylococcus* sp., *Streptococcus* sp., *Actinomyces* sp., and *Mycobacterium* sp.

One of the most renowned terpenoids compounds with anti-microbial activity is fusidic acid, originally derived from the fungal species *Fussidium coccineum*. This compound functions as a bacteriostatic antibiotic, inhibiting bacterial protein synthesis. Currently, it is employed in the form of ointments and creams to address bacterial infections of hair follicles, sweat glands, impetigo, and staphylococcal infections. Scalarane-type sesterpenoids isolated from the fungal species *N. nambi* have undergone testing for their anti-microbial activity. These compounds demonstrated efficacy against *S. aureus*, comparable to the potency of kanamycin, an aminoglycoside antibiotic [58,59]. The growth-inhibitory effects on *S. aureus* strains are demonstrated by psatyrins, diterpenoids found in *Psathyrella candolleana* [60]. Lagopodin B, a benzoquinone sesquiterpenoid, exhibits antibacterial properties against Gram-positive bacteria by acting as a Michael acceptor for thiol groups of bacterial proteins. However, it does not display inhibitory activity against Gram-negative bacteria. This variance in activity may be attributed to the distinct accessibility of the compound to bacterial proteins, influenced by the presence of an outer membrane in the structure of pathogens belonging to this group [61]. The sesquiterpenoid compounds stereumamides A and D, as well as stereostrein Q, identified in the species *S. hirsutum*, demonstrated inhibitory effects on the growth of *S. aureus*, with MICs of 50 µg/mL [20].

The meroterpenoid (Z)-4-hydroxy-3-(3-hydroxy-3-methylbut-1-en-1-yl)benzoic acid, isolated from *Montagnula donacina* at a concentration of 128 µg/mL, exhibited an anti-microbial effect on *S. aureus*. Coprinol, a cuparane-type terpenoid, exhibits activity against multidrug-resistant Gram-positive bacterial strains. Meanwhile, pleuromutilin extracted from *Clitopilus passackerianus* serves as a substrate for the synthesis of the antibiotic retapamulin. Retapamulin is employed in bacterial skin infections and demonstrates the ability to inhibit bacterial protein synthesis by binding to the 50S subunit of ribosomes [62].

The anti-bacterial action mechanisms of fungal terpenes remain incompletely elucidated. In a screening study assessing the affinity of fungal compounds for bacterial proteins, known as the targets of antibiotics, a lanostane-type triterpenoid found in the species *Jahnoporus hirtus* and *Albatrellus flettii* was analyzed. The probable mechanisms of action for this compound include binding to penicillin-binding protein (PbP1a), d-alanyl-d-alanine synthetase (Ddl), topoisomerase IV, and dihydrofolate reductase. Enokipodins A-D, isolated from *F. velutipes*, and ganomycins A and B found in *G. lucidum* showed high affinity for alanine racemase and Ddl, suggesting potential mechanisms for their anti-microbial action [63].

Dermatomycoses rank among the most prevalent dermatological diseases affecting the population, with primary pathogenic strains including *Candida albicans*, *Trichophyton rubrum*, and *Microsporum canis*.

Drimenol, a sesquiterpene isolated from the fungus *Termitomyces* sp., demonstrates anti-fungal activity against *C. albicans* and *Penicillium notatum*. Moreover, it exhibits antibacterial efficacy against *S. aureus* and *Pseudomonas aeruginosa* [64]. Sesquiterpenoids from the *Sanghuangporus* sp. revealed modest anti-microbial activity, inhibiting the growth of *Micrococcus luteus* and *S. aureus* strains. These compounds also exhibited indirect anti-fungal properties against *Mucor hiemalis*, a parasitic species causing zygomycosis in humans [65]. Leptosporin C, a triterpenoid found in *Laetiporus sulphureus*, is one of its inhibitory compounds [66]. Additionally, the diterpenoids pyristriatins A and B extracted from *Cyathus* cf. *striatus* demonstrated anti-microbial and anti-fungal activities, inhibiting the growth of *S. aureus* with MIC values ranging from 8.3 to 16.7 µg/mL [52,67]. The sesquiterpenoids rhodocoranes H and I, discovered in *Rhodotus palmatus*, exhibit anti-microbial properties against *S. aureus* [68].

Promising outcomes have been achieved for many of the previously mentioned compounds in terms of their anti-microbial and anti-fungal activities, providing a basis for advancing knowledge in this field.

### 5.3. Anti-Cancer Effects

Skin cancers rank among the most prevalent human cancers, with metastatic basal cell carcinoma, squamous cell carcinoma, and melanoma being commonly diagnosed types. The incidence and mortality rates of these diseases continue to escalate annually, prompting an intensive search for novel compounds with potential therapeutic efficacy. Numerous fungal terpenes have demonstrated anti-cancer activities, operating through diverse mechanisms of action.

Ganoderic acid DM, a triterpenoid isolated from *G. lucidum*, exhibits significant properties in the realm of cancer therapy. Its anti-proliferative and anti-metastatic effects have been validated in melanoma cell lines 1359-mel, J3, HT-144, DM-331, and B16. Similarly, it has demonstrated these properties against prostate and breast cancer cells. The mechanism of action involves initiating apoptosis and autophagy in tumor-transformed cells. The compound induces cell cycle arrest at the G1 level, impacts signaling pathways, and influences the expression of proteins associated with cellular degradation processes. Ganoderic acid DM upregulates the levels of pro-apoptotic molecules Apaf-1 and Bax while downregulating the synthesis of apoptosis-inhibiting proteins Mcl-1 and Bcl-2. Additionally, it induces the activation of caspase 3, an effector enzyme crucial in the process of programmed cell death. The triterpenoid also enhances the expression of autophagy-inducing factors LC3 and Berlin-1. Studies have demonstrated that these processes can stimulate the immune system against cancer cells. Research conducted on melanoma cells suggests that this compound elevates the presentation of tumor antigens via HLA molecules, leading to increased expression. Furthermore, it enhances the recognition of transformed cells by CD4+ T cells [69].

Other compounds within the triterpenoid group identified in *G. lucidum* show cytotoxicity against cancer cells. Ganoderic acid A and ganodermanontriol exhibit anti-proliferative effects, induce apoptosis, and inhibit cell adhesion and migration. Ganoderic acid T induces programmed cell death by arresting the cell cycle in the G1 phase and inhibiting cell proliferation. Furthermore, lucidenic acids A, B, and N reduce the migration of transformed cells. Ganoderic acid T, Jc, and ganoderiol F have demonstrated well-established cytotoxic effects against melanoma cells [70].

Ophiobolin A, a sesterpenoid identified in fungal species of the *Bipolaris* genus, affects mitochondrial function, as demonstrated in studies evaluating its anti-cancer potential against A375 melanoma cells. It induces disruptions in the mitochondrial membrane potential, resulting in an elevation of reactive oxygen species (ROS) levels. This, in turn, activates caspase 3, induces alterations in the concentrations of Bax and Bcl-2 factors, ultimately leading to cell apoptosis [71].

Anti-cancer activity associated with mitochondrial dysfunction is also demonstrated by GL22, a triterpene isolated from *Ganoderma leucocontextum*. Its mechanism of action is based on the inhibition of fatty acid binding protein (FABP), which is overexpressed in certain cancers and is further correlated with their level of aggressiveness. The blockade of FABP inhibits the transport of fatty acids, resulting in a decrease in the synthesis of cardiolipin—a phospholipid produced in mitochondria. This compound regulates the permeability of mitochondrial membranes and is responsible for the proper functioning of the respiratory chain. The reduction of cardiolipin synthesis by GL22 leads to mitochondrial dysfunction and, consequently, cell death [72].

Grifolin isolated from *Albatrellus confluens*, exerts cytotoxic effects by inhibiting the ERK1/2 signaling pathway, resulting in cell cycle inhibition at the G1 level. Similar to the previously mentioned fungal terpenoids, it influences Bax and Bcl-2 levels, cytochrome C release, and induces an increase in caspase 3, leading to cell apoptosis [73,74].

Fungal terpenes and their derivatives have demonstrated the capacity to elicit anti-cancer effects through various distinct mechanisms of action. Consequently, certain terpene compounds may present innovative avenues in the treatment of skin cancer, either by targeting alternative aspects of the disease or by serving as adjuncts to established therapeutic modalities.

To evaluate the anti-cancer properties of triterpenoid acid extracted from *G. lucidum*, mice were exposed to a carcinogenic compound. The exposed group concurrently received 20-hydroxylucedic acid N at a dose of 85 nmol for a duration of 20 weeks. A significantly lower percentage of mice with developed tumors and a reduced number of tumors per mouse were observed in this group compared to the control group [75]. Studies were conducted using a mouse model of melanoma. Tissues were treated with *Cordyceps taii* extract, leading to the observed inhibition of tumor growth and necrosis in tumor cells. The principal active constituent within the utilized extracts is helvolic acid, classified as a triterpenoid acid [76].

Fungal terpenes can act as adjuncts in cancer therapy by influencing the immune system. Numerous clinical studies involving human subjects have been conducted, administering *G. lucidum* extracts to cancer patients. In one study, participants received 1.8 g of the extract thrice daily for a duration of 6–12 weeks. Following this period, increased natural killer (NK) cell and lymphocyte activity were observed. Similar outcomes were noted in another study involving 68 patients who received extract for 12 weeks, exhibiting increased NK cell and T lymphocyte activity, as well as an impact on the CD4/CD8 ratio. In a separate investigation involving 34 patients treated with the extract for 12 weeks, inflammatory cytokine levels were measured. The results revealed a significant decrease in TNF-α and IL-1 levels, alongside an increase in IL-2, IL-6, and IFN-*γ* in the plasma of the study subjects [70]. The impact on the immune system could potentially stem from the presence of triterpenes and their derivatives exhibiting established anti-inflammatory and immunomodulatory properties in the extracts. However, further research is necessary to substantiate this hypothesis.

### 5.4. Inhibition of Tyrosinase

Skin pigmentation disorders comprise a category of skin conditions linked to disrupted melanin synthesis or transport. Discoloration can manifest across the entire or partial body surface or appear as localized spots. In instances of widespread alterations, primary factors include hormonal shifts, genetic predispositions, or deficiencies in vitamins. Spot and localized lesions typically stem directly from disruptions in melanogenesis. The most frequently recognized ones include coffee-au-lait spots, freckles, post-inflammatory spots and lentigines. One of the promising forms of therapy is the inhibition of the activity of tyrosinase—a key enzyme involved in the synthesis of melanin. Many fungal terpenes have significant blocking abilities.

The biosynthetic pathway for melanin formation in different bioforms is mainly regulated by tyrosinase in two different oxidation reactions. Basically, tyrosinase catalyzes the hydroxylation of tyrosine to dihydroxyphenylalanine (DOPA) and the oxidation of DOPA to DOPAquinone, which is further converted into eumelanin (a brown-black pigment) or pheomelanin (a yellow-red pigment), depending on various physiological conditions. Thus, tyrosinase inhibition is the most common method of achieving skin whitening [77]. Numerous fungi show tyrosinase inhibitory activity determined by the activity of terpene-structured compounds.

Lanostane-structured triterpenoids: hispindic acid A and B isolated from the fruiting bodies of *Inonotus hispidus*, whose structure was elucidated by extensive spectroscopic analysis (NMR and HRMS), were evaluated for their ability to activate melanogenesis and tyrosinase in B16 melanoma cells. Hispindic acid A showed stronger melanogenesis and tyrosinase activating abilities in the cells tested than the positive control, 8-methoxypsoralen, used as a positive control at a concentration of 50 mmol/L [78]. Extracts of *I. obliquus*, obtained using solvents of different polarity, were evaluated for their ability to inhibit tyrosinase, the rate-limiting enzyme in melanogenesis. This study confirmed that extracts obtained with petroleum ether and *n*-butanol had an inhibitory effect on tyrosinase, while the ethyl acetate extract had an activating effect. In cell assays, betulin and trametenolic acid (Figure 1) decreased tyrosinase activity and melanin content, whereas inotodiol and lanosterol (Figure 1) significantly increased tyrosinase activity and melanin content, showing AC_50_ values of 9.74 and 8.43 µM, respectively. In contrast, 3β,22,25-trihydroxy-lanosta-8-ene, had little or no effect on tyrosinase. Betulin showed non-competitive inhibition with K_I_ = K_IS_ of 0.4 µM of tyrosinase activity, IC_50_ of 5.13 µM and being more effective than kojic acid (6.43 µM). Trametenolic acid showed a mode of mixed inhibition with K_I_ of 0.9 µM, K_IS_ of 0.5 µM and IC_50_ of 7.25 µM [79].

The presence of terpenoid-structured metabolites was confirmed in extracts from selected *Trametes* species: *T. gibbosa, T*. *versicolor*, and *T. hirsuta*, obtained from the mycelium and fruiting bodies of these species. These extracts were analyzed for their ability to inhibit tyrosinase activity. The analysis revealed that the mycelial extract of *T. gibbosa* was a significantly more effective inhibitor of tyrosinase activity than kojic acid, with a concentration effectiveness of 40.9%. Chemical analysis demonstrated strong synergistic effects among triterpenes, sugars, and polyphenols [80].

Compared to other Basidiomycota, *G. lucidum* is a species with outstanding skin whitening properties. A compound named ganodermanondiol, which has a pentacyclic triterpenoid structure, was isolated from an ethanol extract obtained from the fruiting bodies of *G. lucidum*. Ganodermanondiol demonstrates anti-pigmentation potential by inhibiting tyrosinase activity and melanin biosynthesis in B16F10 melanoma cells. This compound affects the expression of tyrosinase, TRP-1, TRP-2, and MITF, leading to a reduction in melanin production. Furthermore, ganodermanondiol influences the MAPK and cAMP signaling pathways involved in the melanogenesis of B16F10 melanoma cells. Tyrosinase, TRP-1, and TRP-2 play key roles in melanin biosynthesis, determining the shape of melanosomes in melanocytes. A study on the effects of ganodermanondiol on melanogenesis showed suppression of tyrosinase, TRP-1, and TRP-2 expression following pretreatment of cells with ganodermanondiol prior to α-MSH hormone stimulation. The process of melanogenesis is regulated by various transcription factors, primarily MITF. In conclusion, ganodermanondiol effectively inhibits the expression of tyrosinase, TRP-1, and TRP-2, through the downregulation of MITF. Studies indicate that the MITF pathway plays a crucial role in the inhibition of melanogenesis by ganodermanondiol. The activation of the melanogenic pathway involving SCF and EDN1 is well-established. A study on the effects of ganodermanondiol on the phosphorylation of MAPK family proteins showed increased phosphorylation of ERK and JNK and decreased phosphorylation of p38. These results suggest that the inhibition of melanogenesis by ganodermanondiol is related to the regulation of MAPK proteins, where phosphorylation of ERK and JNK contributes to this process, and phosphorylation of p38 is inhibited by ganodermanondiol [53,54].

In vitro and in vivo studies involving humans have been conducted to explore the use of *Poria cocos* (syn. *Wolfiporia cocos*) extracts in treating skin discoloration. In the initial phase, the extract’s ability to inhibit tyrosinase in melanoma cells was demonstrated. This was followed by a randomized, double-blind clinical trial involving 40 women aged 20 to 30. The treatment group applied a 2% cream, using 2 g on each cheek, both in the morning and evening for four weeks. Spectrophotometric examinations of both cheeks were conducted after 2 and 4 weeks. The results showed significant skin lightening, which varied with the duration of application. This observed effect may be attributed to the presence of terpenes in *P. cocos* extracts, which have a proven ability to inhibit tyrosinase [81].

### 5.5. Photoprotective Properties

Sunburns result from inflammatory damage to the skin induced by overexposure to UV radiation, characterized by manifestations such as redness, pain, and swelling. Beyond short-term discomfort, sunburns can elicit enduring negative health effects, particularly with recurrent and prolonged sun exposure. These repercussions include hyperpigmentation, skin aging, and DNA damage, which may ultimately contribute to the development of cancer. Certain fungal terpenes and their derivatives exhibit photoprotective properties in human keratinocytes and demonstrate regenerative capabilities.

A study was conducted on mice exposed to UVB radiation (for 30 min) to induce skin damage. Subsequently, some mice were administered oral and topical extracts of the fungal species *P. cocos* for a two-week period. The intervention group exhibited significantly accelerated healing of skin lesions compared to the control group, manifesting as a reduction in wounds, erosions, and redness. This observed effect is likely attributable to the diminished membrane permeability in mast cells, the inhibition of pro-inflammatory cytokine synthesis, and the mitigation of oxidative stress. Moreover, evidence suggests a potential link between these outcomes and the impact of the extract’s active compounds on the gut microbiome-skin axis. UVB radiation has been shown to disrupt the gut microbial flora, leading to compromised immunity and increased symptoms. Conversely, *P. cocos* extract has demonstrated the capability to restore bacterial balance, potentially contributing to expedited recovery and enhanced resistance to damage. The primary active compounds within the extract are believed to be terpenes, characterized by their elevated concentration and established anti-inflammatory and antioxidant properties [82].

Table 1 presents a summary of the biological activities of terpenes and terpenoids derived from fungi and their verified applications to the skin.

## 6. Methods

A well-organized search strategy is crucial for accurately defining appropriate search terms and identifying the topical databases needed to gather a satisfactory amount of scientific literature. Eligible literatures were selected based on predefined inclusion and exclusion criteria. These criteria were established to exclude publications that were either not empirical in nature or were not classified as documents or guidelines, such as commentaries, letters, and book reviews. We established that the spectrum of main interests must include fungal terpenes and terpenoids, in the fields of phytochemistry, mycology, cosmetology, and pharmacology, in the broadest sense. The databases searched for this review included SCOPUS, PubMed/MEDLINE, Web of Science, Wiley Online Library, Taylor and Francis Online, Google Scholar, REAXYS Database, Science Direct/ELSEVIER, and EBSCO Discovery Service. These databases were systematically searched for articles published between 1950 and 2023.

## 7. Conclusions

In summary, the investigation into fungal terpenes and terpenoids and their therapeutic potential in relation to skin diseases has yielded valuable insights. Numerous studies on terpene compounds, encompassing their properties, mechanisms of action, and potential applications in treating specific diseases, elucidate the intricate nature of these compounds. Remarkably, numerous isolated terpenes and their derivatives have exhibited notable anti-inflammatory and anti-microbial activities, with some surpassing or matching the efficacy of current treatments for inflammatory and infectious skin diseases.

Exploration into the anti-cancer and antioxidant properties of fungal terpenes and terpenoids suggests a promising avenue for advancing skin cancer prevention and treatment. Moreover, certain terpenoids displaying inhibitory effects on tyrosinase offer opportunities for addressing skin pigmentary disorders and cancers linked to melanogenesis dysfunctions. The observed photoprotective and regenerative effects of specific isolated compounds on the skin underscore the diverse therapeutic potential of fungal terpene compounds.

The inclusion of a discussion on selected skin diseases, along with insights from clinical and in vivo studies, provides a practical context for understanding the applications and efficacy of fungal terpenoids in real-world scenarios. These findings not only contribute to the scientific understanding of terpenes but also propose avenues for developing novel treatments and interventions for various skin conditions. Ongoing research in this field has the potential to revolutionize current approaches to skin disease management, paving the way for innovative therapeutic strategies.

## Figures and Tables

**Table 1:** Biological activities of fungal-derived substances and their confirmed applications to the skin.

Terpenes/Terpenoids	Species	Activity	Activity Description	Reference
Inotodiol	*Inonotus obliquus*	Anti- inflammatory	Decrease of IL-1, IL-6 and IL-8 levels	[11]
		Tyrosinase activation	Increase of melanin level	[79]
Pholiol F	*Pholiota populnea*	Anti- inflammatory	COX-2 and 5-LOX inhibition—decrease of leukotrienes and prostaglandins secretion	[41]
Lepiotaprocerins	*Macrolepiota procera*	Anti- inflammatory	Decrease of NO synthesis, decrease of PGE2, TNF-α and IL-1β levels	[42]
Dehydroabietanoic acid	*Phellinus pini*	Anti- inflammatory	Inhibition of NO release	[44]
Phellibarins B, C	*Phellinus* *rhabarbarinus*	Anti- inflammatory	Inhibition of NO release	[44]
Gilvinin A	*Phellinus* *rhabarbarinus*	Anti- inflammatory	Inhibition of NO release	[44]
Igniarene B	*Phellinus igniarius*	Anti- inflammatory	iNOS inhibition	[44]
Tricholopardin A	*Tricholoma* *pardinum*	Anti- inflammatory	Inhibition of NO production, decrease of PGE2 TNF-α and IL-1β levels	[45]
Ganodermanontriol	*Ganoderma lucidum*	Anti- inflammatory	Decrease of NO, TNF-α, IL-1β, and IL-6 levels, inhibition of NF-κB and MAPK pathway, decrease of TLR4 and MyD88 expression	[47]
		Anti-cancer	Anti-proliferative effect, apoptosis of cancer cells, inhibition of cell adhesion and migration	[70]
Ganoderiols A, D, F	*Ganoderma lucidum*	Anti- inflammatory	Decrease of NO, TNF-α, IL-1β, and IL-6 levels	[47]
Poricoic acid GM	*Wolfiporia cocos*	Anti- inflammatory	Decrease of NO, IL-1β, IL-6, TNF-α, and PGE2 levels, inhibition of NF-κB pathway, iNOS inhibition, decrease of COX-2 expression	[48]
Ganoresinoids	*Ganoderma* *resinaceum*	Anti- inflammatory	Decrease of IL-1β, IL-6, and TNF-α levels, inhibition of NF-κB and MAPK pathway, decrease of TLR4 expression	[49]
Crinipellins F, G	*Crinipellis* sp.	Anti- inflammatory	Decrease of CxCl1, CxCl11, CCL3, IL-6, IL-8, TNF-α, CCL2 and COX-2 synthesis, inhibition of NF-κB pathway	[50]
Psatyrins	*Psathyrella* *candolleana*	Antibacterial	*Stapylococcus aureus*	[60]
Lagopodin B	*Coprinopsis cinerea*	Antibacterial	Gram-positive bacteria	[61]
Stereumamides A, D	*Stereum hirsutum*	Antibacterial	*Stapylococcus aureus*	[20]
Stereostrein Q	*Stereum hirsutum*	Antibacterial	*Stapylococcus aureus*	[20]
(Z)-4-hydroxy-3-(3-hydroxy-3-methylbut-1-en-1-yl)benzoic acid	*Montagnula donacina*	Antibacterial	*Stapylococcus aureus*	[62]
Drimenol	*Termitomyces* sp.	Antifungal Antibacterial	*Candida albicans*, *Penicilium notatum* *Stapylococcus aureus*, *Pseudomonas* *aeruginosa*	[64]
Leptosporin C	*Laetiporus sulphureus*	Antifungal	*Mucor hiemalis*	[66]
Pyristriatins A, B	*Cyathus* cf. *striatus*	Antibacterial	*Stapylococcus aureus*	[67]
Rhodocoranes H, I	*Rhodotus palmatus*	Antibacterial	*Stapylococcus aureus*	[68]
Ganoderic acid DM	*Ganoderma lucidum*	Anti-cancer	Anti-proliferative and anti-metastatic effect, apoptosis and autophagy of cancer cells, increased recognition of cancer cells by CD 4+ T lymphocytes	[69]
Ganoderic acid A	*Ganoderma lucidum*	Anti-cancer	Anti-proliferative effect, apoptosis of cancer cells, inhibition of cell adhesion and migration	[70]
Ganoderic acid T	*Ganoderma lucidum*	Anti-cancer	Anti-proliferative effect, apoptosis of cancer cells, cytotoxicity	[70]
Lucidenic acids A, B, N	*Ganoderma lucidum*	Anti-cancer	Inhibition of cell migration	[70]
Ganoderiol F	*Ganoderma lucidum*	Anti-cancer	Cytotoxicity	[70]
Ophiobolin A	*Bipolaris* sp.	Anti-cancer	Mitochondrial dysfunction and apoptosis of cancer cells	[71]
GL22	*Ganoderma* *leucocontextum*	Anti-cancer	FABP inhibition, mitochondrial dysfunction and apoptosis of cancer cells	[72]
Grifolin	*Albatrellus confluens*	Anti-cancer	Cytotoxicity and apoptosis of cancer cells	[73,74]
Trametenolic acid	*Inonotus obliquus*	Tyrosinase inhibition	Decrease of melanin level	[79]
Hispindic acids A, B	*Inonotus hispidus*	Tyrosine activation	Melanogenesis activation	[78]
Ganodermanondiol	*Ganoderma lucidum*	Tyrosinase inhibition	Decrease of melanin level	[53,54]

## Data Availability

No new data were created.
